# Predicting Longitudinal Outcomes of Alzheimer’s Disease via a Tensor-Based Joint Classification and Regression Model

**Published:** 2020

**Authors:** Lodewijk Brand, Kai Nichols, Hua Wang, Heng Huang, Li Shen, Alzheimer’s Disease Neuroimaging Initiative

**Affiliations:** Department of Computer Science, Colorado School of Mines, Golden, CO 80401, USA; Department of Computer Science, Colorado School of Mines, Golden, CO 80401, USA; Department of Computer Science, Colorado School of Mines, Golden, CO 80401, USA; Department of Electrical and Computer Engineering, University of Pittsburgh, Pittsburgh, PA 15206, USA; Department of Biostatistics Epidemiology and Informatics, University of Pennsylvania, Philadelphia, PA 19104, USA

**Keywords:** Alzheimer’s Disease, Biomarker Identification, Multi-Modal, Regression, Classification, the Alternating Direction Method of Multipliers

## Abstract

Alzheimer’s disease (AD) is a serious neurodegenerative condition that affects millions of people across the world. Recently machine learning models have been used to predict the progression of AD, although they frequently do not take advantage of the longitudinal and structural components associated with multi-modal medical data. To address this, we present a new algorithm that uses the multi-block alternating direction method of multipliers to optimize a novel objective that combines multi-modal longitudinal clinical data of various modalities to simultaneously predict the cognitive scores and diagnoses of the participants in the Alzheimer’s Disease Neuroimaging Initiative cohort. Our new model is designed to leverage the structure associated with clinical data that is not incorporated into standard machine learning optimization algorithms. This new approach shows state-of-the-art predictive performance and validates a collection of brain and genetic biomarkers that have been recorded previously in AD literature.

## 1. Introduction

Alzheimer’s disease (AD) is a neurodegenerative disorder that has serious mental and financial consequences for those affected and their families. AD is characterized by progressive declines of memory and cognitive capabilities. According to the *Alzheimer’s Association*^1^ 5.7 million people in the United States are currently suffering from AD-related dementia. In 2018 alone, the total financial cost associated with health care, long-term care, and hospice services for patients suffering from dementia was estimated to be $277 billion. It is forecasted that by 2050, the number of people suffering from AD will surpass 13.8 million. Furthermore, the *Alzheimer’s Association* emphasizes that early detection and diagnosis of individuals with AD could save up to $7.9 trillion in associated medical costs. With the projected increase in individual hardship and financial burden caused by AD, it is essential that the scientific community develop computational methods for early diagnosis and treatment of AD.

A central research component, designed to assist in early identification of dementia, has focused on discovering characteristic biomarkers that are closely associated with the development of AD. This branch of research has been driven by the successful development and deployment of a variety of non-invasive clinical observations such as positron emission tomography (PET), magnetic resonance imaging (MRI) scans, and genetic analysis through the identification of single nucleotide polymorphisms (SNPs). By way of public-private partnerships, such as the Alzheimer’s Disease Neuroimaging Initiative (ADNI),^39^ clinical data from each of theses modalities have been made publicly available to the scientific community. Through the effective analyses of these AD data sources, we are able to build models that have the potential to help clinical researchers narrow down the array of phenotypic and genetic measures that are predictive of cognitive decline. Given the complexity and size of these clinical datasets, there has been a concerted effort to design new machine learning methods to assist in the discovery of AD-related biomarkers.

In recent years, various computational methods^18,27,40,41^ have been proposed to identify biomarkers associated with AD. Although these methods have shown good predictive performance, they only incorporate clinical data that is collected at a single time-point. Since these approaches rely on a single point in time, they are unable to identify longitudinal patterns found across patient data. Recent works^3,15,33,34^ explored using longitudinal data to predict an AD diagnosis, which validated that specific regions of the brain (derived from neuroimaging modalities) are the most useful for diagnosing AD over time.

With the above recognitions, in this work we aim to develop a principled approach to incorporate *longitudinal* data from *multiple* data sources that the ADNI provides. Through extensive empirical studies, our new approach has shown great promise in predicting cognitive scores, diagnoses and identifying AD-relevant genetic and phenotypic biomarkers. Specifically, we present the following:

- A principled strategy for incorporating tensor data (*e.g*. longitudinal) collected from multiple data sources, which leads to a new objective that is able to combine multimodal longitudinal clinical data of various modalities to simultaneously predict the cognitive scores and diagnoses of the participants in the ADNI cohort.
- An effective optimization algorithm, using the multi-block alternating direction method of multipliers, to optimize the proposed objective.
- A collection of phenotypic biomarkers, some of which have been shown by previous research to be predictive of cognitive decline, identified by our model.

## 2. Methods

In this manuscript, we write tensors as cursive uppercase letters (***A***, ***B***, ***C***,...), matrices as bold uppercase letters (**A**, **B**, **C**,...), vectors as bold lowercase letters (**a**, **b**, **c**,...), and scalars as lowercase letters *(a, b, c*,...). Given a matrix **M**, its *i*-th row and *j*-th column are denoted as ***m***^*i*^ and ***m***_*j*_ respectively. We define the Frobenius norm of the *m* × *n* matrix **A** as $\|A{\|}_{F}=\sqrt{\sum_{i=1}^{m}\sum_{j=1}^{n}{\left|a_{ij}\right|}^{2}}$.

**Fig. 1.**
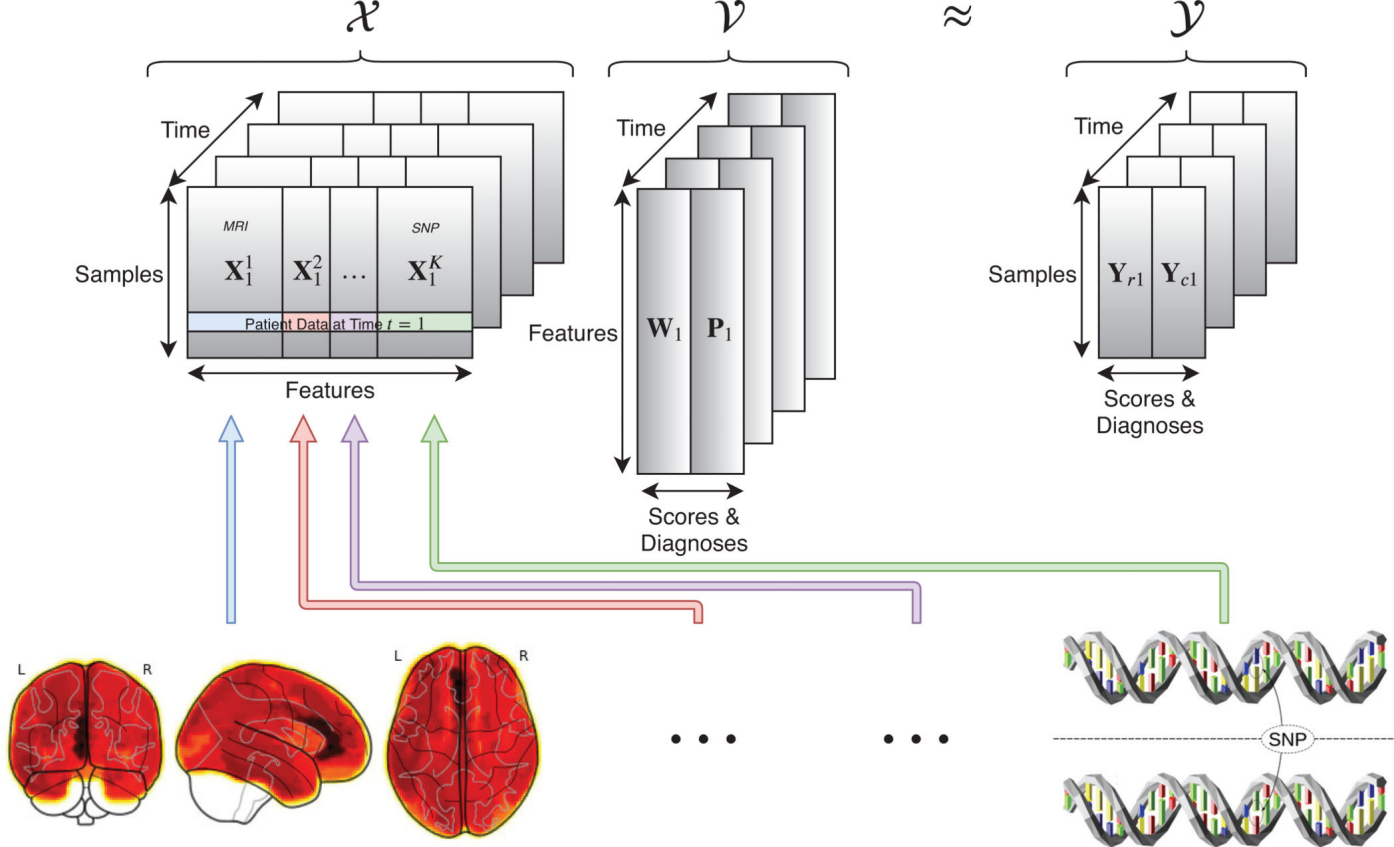
Visualization of the input (*X*), coefficient (*V*) and output (*Y*) tensors. In each time-point of *X* the *K* modalities (MRI, SNP, *etc*.) are explicitly defined to facilitate the calculation of the group *l*_1_-norm. The goal of the proposed method is to learn a joint model *V* that can effectively map *X* to the cognitive scores and diagnoses encoded in *Y*.

The input imaging features are represented by the tensor: $X=\left\{X_{1},X_{2},\ldots ,X_{T}\right\}\in {\mathbb{R}}^{n\times d\times T}$ Each **X**_*t*_ represents the input observations for *n* patients with *d* features at a given time *t.* Each **X**_*t*_ can be further broken down into *K* modalities: ${\left\{X_{t}^{j}\right\}}_{j=1}^{K}$. The output diagnoses and cognitive scores are represented by another tensor:$y=\left\{Y_{1},Y_{2},\ldots ,Y_{T}\right\}\in {\mathbb{R}}^{n\times c\times T}$. Each $Y_{t}=\left[Y_{rt}Y_{ct}\right]$ is a concatenation of the cognitive scores (for regression) and diagnosis (for classification) for *n* patients at time *t*. The goal of our proposed new machine learning model is to learn a joint regression and classification model represented by the tensor $V=[WP]:V=\{\left[W_{1}P_{1}\right],[W_{2}P_{2}],\ldots ,\left[W_{T}P_{T}\right]\}\in {\mathbb{R}}^{d\times c\times T}$ where $W_{t}\in {\mathbb{R}}^{d\times c_{r}}$ and $P_{t}\in {\mathbb{R}}^{d\times c_{e}}$ are the learned coefficient matrices for the respective regression and classification tasks. The input X output Y, and learned coefficient V tensors are illustrated in Fig. 1.

### 2.1. The Longitudinal Joint Learning Model

A key idea behind our approach is to perform the regression and classification tasks at the same time. Joint regression and classification can help discover more robust patterns than those discovered when classification and regression are performed separately.^3,31,35^ In order to link the regression and classification tasks, following the large body of previous works^3,31,35^ we introduce the following regularized joint learning model:

(1) $$\underset{W,P}{\min}L_{r}(W)+L_{c}(P)+R(V),$$

where $L_{r}$ and $L_{c}$ are the prescribed loss functions associated with the regression and classification tasks respectively. Here the regularization function R(V) is applied to the matrix unfolded from tensor V, *i.e*., we construct $V\in R^{d\times cT}$ by taking the (**W**_*t*_, **P**_*t*_) matrix pairs at each time-point and joining them along their columns.^33,34^ This joint regularization scheme in Eq. (1) is designed to identify features in X that are predictive of both clinical diagnoses and cognitive scores. This approach *reasonably assumes* that there exists a relationship between the classification and regression tasks. For example, if a patient does poorly on a given cognitive test then they are more likely to be diagnosed with AD. Regularizing the joint coefficient matrices (W,P) allows us to discover biomarkers that are strongly associated with the two related tasks. We design the regularization function R(V) as following.

First, in order to associate the longitudinal imaging and genetic markers to predict cognitive scores and diagnoses over time, we apply the widely used *l*_2,1_-norm^20,32^ to the unfolded coefficient matrix $V:\|V{\|}_{2,1}=\sum_{i=1}^{d}{\left\|v^{i}\right\|}_{2}$.

Second, as we combine *K* different modalities (MRI, SNP, FreeSurfer, *etc*.) together, it is critical for our model to differentiate the impact that each modality has on the joint model. In order to capture the impact of each modality, we leverage the group *l*_1_-norm (*G*_*1*_ -norm):^3,35–37^ $\|V{\|}_{G_{1}}=\sum_{j=1}^{K}{\left\|V^{j}\right\|}_{2}$, where **V**^*j*^ is a matrix constructed of the rows in *V* that corresponds to the *j-th* modality inX.

Finally, we know that as AD develops, many cognitive measures are related to one another within the same modality. In order to account for this inter-modal relationship, we leverage the trace norm regularization^21,24,33,34,38^ of $V:\|V{\|}_{*}=\sum \sigma_{i}(V)$, where σ_*i*_ (**V**) are the singular values of **V**.

Bringing together these three regularizations, we present our new objective as following:

(2) $$\begin{array}{l} \min_{V}J=\sum_{t=1}^{T}\left[{\left\|X_{t}W_{t}-Y_{rt}\right\|}_{F}^{2}\right]+\sum_{t=1}^{T}\sum_{i=1}^{n}\sum_{k=1}^{c_{e}}\left[{\left(1-\left(x^{it}p_{kt}+b_{kt}\right)y_{ikt}\right)}_{+}\right]\\ +\gamma_{1}\|V{\|}_{2,1}+\gamma_{2}\|V{\|}_{G_{1}}+\gamma_{3}\|V{\|}_{*}, \end{array}$$

where the first term is the multivariate regression loss at each longitudinal time-point; and the second term represents the loss of *c*_*C*_ *× T* one-vs-all multi-class support-vector machine (SVM) penalized via the hinge-loss, where $y_{ikt}\in \{-1,1\}$ is the class label associated with *i*-th patient at time *t,* and $b_{kt}$ is the bias associated with the (*k* × *t*)-th SVM. The notation (·)_+_ is defined as (a)_+_ = max(0, *a*).

### 2.2. The Solution Algorithm Using the Multi-Block ADMM

While the objective of our new method in Eq. (2) is clearly and reasonably motivated, all its terms are dependent on V. Thus, it is difficult to optimize this objective in general. To solve the proposed objective, we derive an efficient iterative algorithm using the multi-block extension^8^ of the alternating direction method of multipliers (ADMM).^2^

The ADMM aims to decouple a larger and more difficult problem into a series of smaller sub-problems that are easier to solve.^2^ An extension to ADMM, known as multi-block ADMM,^8^ is designed to extend the ADMM framework to optimize functions of the following form:

(3) $$\begin{array}{l} \underset{{}_{x_{i}}}{\min}\;f_{1}\left(x_{1}\right)+f_{2}\left(x_{2}\right)+\cdots +f_{K}\left(x_{K}\right),\\ \;\text{subject}\;\text{to}\;\;E_{1}x_{1}+E_{2}x_{2}+\cdots +E_{K}x_{K}=c. \end{array}$$

Equation. (3) can be solved by minimizing the following unconstrained objective:^2,8^

(4) $$L_{\mu }\left(x_{1},x_{2},\ldots ,x_{k},y\right)=\sum_{k=1}^{K}f\left(x_{k}\right)+\frac{\mu }{2}{\left\|\sum_{k=1}^{K}E_{k}x_{k}-c+\frac{1}{\mu }y\right\|}_{2}^{2},$$

where *y* is a Lagrangian multiplier and *μ >* 0 is a constant. The objective in Eq. (4) can be solved by the following iterative procedure that updates each *x*_*k*_ (primal) and the Lagrangian variable *y* (dual):

(5) $$\{\begin{array}{l} x_{1}^{t+1}\leftarrow \arg\min_{x_{1}}L_{\mu }\left(x_{1}^{t},x_{2}^{t},\cdot ,x_{K}^{t}\right),\\ \cdots \\ x_{K}^{t+1}\leftarrow \arg\min_{x_{K}}L_{\mu }\left(x_{1}^{t+1},x_{2}^{t+1},\ldots ,x_{K}^{t}\right),\\ y^{t+1}=y^{t}+\mu \left(\sum_{k=1}^{K}E_{k}x_{k}-c\right),\\ \mu^{t+1}=\rho \mu^{t}, \end{array}$$

where *ρ >* 1 is a constant. The process described above in Eq. (5) is repeated until the algorithm converges. In order to decouple the terms containing V in Eq. (2), we introduce four new variables and a set of corresponding equality constraints as following:

(6) $$\begin{array}{l} \min_{V}J=\sum_{t=1}^{T}\left[{\left\|X_{t}W_{t}-Y_{rt}\right\|}_{F}^{2}\right]+\sum_{t=1}^{T}\sum_{i=1}^{n}\sum_{k=1}^{c_{c}}\left[{\left(y_{ikt}e_{ikt}\right)}_{+}\right]\;\\ +\gamma_{1}\|F{\|}_{2,1}+\gamma_{2}\|G{\|}_{G_{1}}+\gamma_{3}\|H{\|}_{*},\\ \text{subject to}\;e_{\mathrm{ikt}}=y_{\mathrm{ikt}}-(x^{\mathrm{it}}p_{\mathrm{kt}}+b_{\mathrm{kt}}),\;\text{F}=\text{V},\;\text{G}=\text{V},\;\text{and}\;\text{H}=\text{V}. \end{array}$$

Since each *y*_*ikt*_ in the second term must be equal to either −1 or 1, we can use the following to move from Eq. (2) to Eq. (6):$1-\left({\text{x}}^{it}p_{kt}+b_{kt}\right)y_{ikt}=y_{ikt}y_{ikt}-\left({\text{x}}^{it}p_{kt}+b_{kt}\right)y_{ikt}=y_{ikt}(y_{ikt}-\left(x^{it}p_{kt}+b_{kt}\right)$. ^22^ Then we can solve Eq. (6) by minizing the following ojective:

(7) $$\begin{array}{l} L_{\mu }\left(V,e_{ikt},F,G,H,\lambda_{ikt},\Sigma ,\Theta ,\Omega \right)=\sum_{t=1}^{T}\left[{\left\|X_{t}W_{t}-Y_{rt}\right\|}_{F}^{2}\right]+\sum_{t=1}^{T}\sum_{i=1}^{n}\sum_{k=1}^{c_{c}}\left[{\left(y_{ikt}e_{ikt}\right)}_{+}\right]\\ +\frac{\mu }{2}\sum_{t=1}^{T}\sum_{i=1}^{n}\sum_{k=1}^{c_{e}}\left[{\left(e_{ikt}-\left(y_{ikt}-\left({\text{x}}^{it}p_{kt}+b_{kt}\right)\right)+\frac{1}{\mu }\lambda_{ikt}\right)}^{2}\right]+\gamma_{1}\|F{\|}_{2,1}+\gamma_{2}\|G{\|}_{G_{1}}+\gamma_{3}\|H{\|}_{*}\\ +\frac{\mu }{2}{\left\|F-V+\frac{1}{\mu }\Sigma \right\|}_{F}^{2}+\frac{\mu }{2}{\left\|G-V+\frac{1}{\mu }\Theta \right\|}_{F}^{2}+\frac{\mu }{2}{\left\|H-V+\frac{1}{\mu }\Omega \right\|}_{F}^{2}, \end{array}$$

where λ_*ikt*_, **Σ**, **Θ**, and **Ω** are the Lagrangian multipliers. The updates for each of the primal variables can be calculated by taking the derivative of Eq. (7), with respect to each of the primal variables, setting the resulting equation equal to zero, and solving for the associated primal variable. Due to space considerations, we will provide the detailed mathematical derivation for each variable in an extended journal version of this paper. The derived parameter updates are provided in Algorithm 1.

## 3. Experiments

### Data.

We downloaded MRI scans, SNP genotypes, and demographic information for 821 ADNI-1 participants. We performed FreeSurfer automated parcellation on the MRI data by following Risacher *et al*.^25^ and extracted mean modulated gray matter measures for 90 target regions of interest. We followed the SNP quality control steps discussed in Shen *et al*.^26^ We also downloaded the longitudinal scores of the participants’ Rey’s Auditory Verbal Learning Test (RAVLT) and their clinical diagnosis: Alzheimer’s disease (AD), mild cognitive impairment (MCI), and healthy control (HC). All the participants with no missing Baseline/Month 6/Month 12/Month 24 MRI measurements, SNP genotypes, and cognitive measures were included in this study, resulting in a set of 412 subjects (79 AD, 190 MCI, 143 HC at Baseline, 86 AD, 180 MCI, 155 HC at Month 6, 111 AD, 155 MCI, 146 HC at Month 12, and 155 AD, 110 MCI, 147 HC at Month 24).

### Settings.

The performance and standard deviation results reported in Table 1 and Table 2 are calculated from *ten* five-fold cross validation experiments applied to X and Y; in-between each cross validation experiment X and Y are randomly shuffled. Each method reported in the following experiments were tuned via a reasonable hyper parameter search to guarantee a fair comparison. The optimal tuning parameters are chosen by the model that provides the best regression or classification performance during a single five-fold cross validation experiment. In choosing the parameters for our new method, we fine tuned the γ parameters, described in Eq. (7), by applying powers of 10 between 10^−5^ and 10^5^ and choosing the best model based on the average multitask performance. Following the search, we achieve the best performance at $\gamma_{1}=.00001,\gamma_{2}=.01,\gamma_{3}=100,\mu =.001$ and *ρ* = 1.2, which we use in all our experiments.

### 3.1. Performance

#### Regression.

We compare our algorithm against multivariate linear regression *(Linear*), *l*_*2*_*-* regularized linear regression *(Ridge*), *l*_1_-regularized linear regression *(Lasso*),^29^ and multi-layer perception regression *(MLP*).^7^ In Table 1, our new method shows superior regression performance when compared to the aforementioned methods. This is likely because our method incorporates information provided by the longitudinal regularizations across both tasks.

**Algorithm 1:** The solution algorithm to optimize Eq. (2).

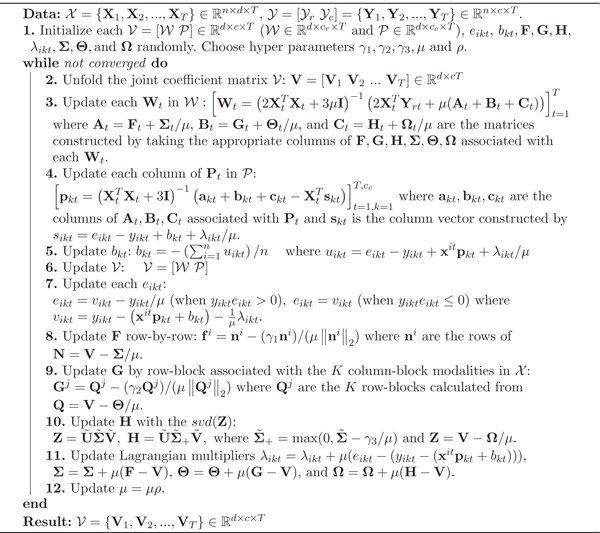

#### Classification.

We report the iterated five-fold cross validation results on the classification task of our method compared to a variety of popular machine learning algorithms for classification in Table 2. We compare our method against logistic regression (*Logistic*), random forest classifier *(RandomForest*), support vector machine using a sigmoid-kernel *(SVM*), k-nearest neighbors classifier *(KNN*), logistic regression with elastic net regularization *(ElasticNet*),^4^ and a linear support vector machine *(LinearSVM*).^13^ Both *ElasticNet* and *LienarSVM* have been used in the past to classify patients with AD vs. HC. From Table 2 we can see that our algorithm shows significant improvement when predicting AD and HC diagnoses. This improvement does not appear to extend to MCI diagnoses, where logistic regression improves upon our model. This disparity is likely because the *c*_*c*_ one-vs-all multi-class SVMs constructed in P are not normalized against one another. Nonetheless outperforms the detection of HC and AD in ADNI participants when compared to the methods in Table 2.

### 3.2 Empirical Convergence

It is well known that the multi-block ADMM approach described in Algorithm 1 does not necessarily converge.^5^ So, in order to determine convergence properties of the proposed algorithm, we perform the following empirical analyses. First, we want to determine whether the initialization of the model has a significant effect on the convergence of Algorithm 1. Second, we want to determine whether our multi-block optimization scheme actually matches the constraints incorporated by the augmented Lagrangian after a reasonable number of iterations.

**Fig. 2.**
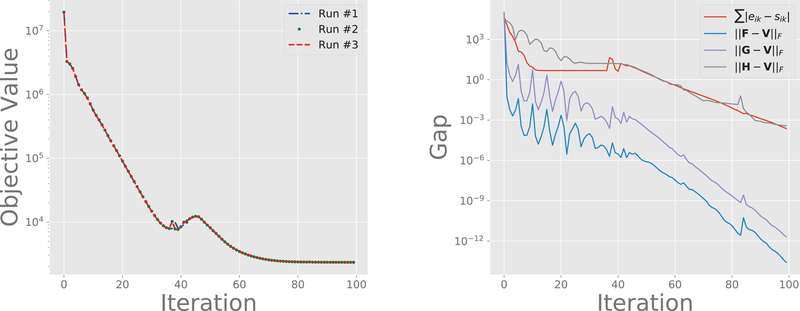
**Left:** The proposed objective in Eq. 7 plotted over one-hundred iterations of Algorithm 1. In each run the primal and dual variables are randomly re-initialized. **Right:** The difference between the introduced variables designed to decouple the terms in Eq. (2).

To analyze the first issue we apply our algorithm to the same dataset three times and plot the objective on the left-hand-side of Fig. 2. This plot shows that, even with random initialization, our algorithm converges to a similar solution after only one-hundred iterations. To analyze the second issue, we plot the difference between the introduced variables *(e*_*ik*_, **F**, **G**, **H**) designed to decouple the original objective in Eq. (2). As can be seen on the right-hand-side of Fig. 2, once the objective has converged the difference between the decoupled variables and the variables that they replaced are within 10^−3^ after one-hundred iterations of the proposed method. The convergence of the overall objective across differently initialized runs, and the eventual gap decrease, provide empirical evidence for the convergence of the proposed multi-block ADMM algorithm.

### 3.3 Biomarker Identification

In addition to predictive performance, our method is easily interpreted and can assist in the identification of AD-related biomarkers.

#### MRI.

**Fig. 3.**
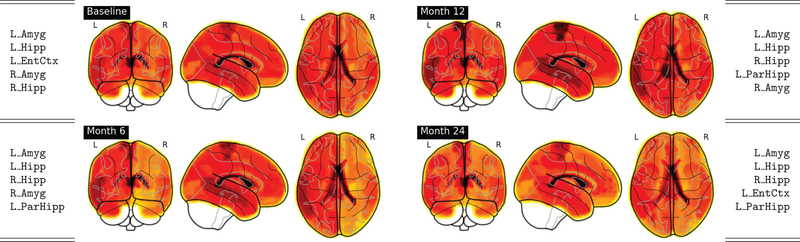
Top-5 ordered biomarkers in the FreeSurfer modality at each time-point. The identified biomarkers, listed on the *far-left* and *far-right,* are ordered from largest coefficient (top) to smallest (bottom) derived from *V*.

In Fig. 3 we plot the magnitudes, derived from V, of coefficients associated with the FreeSurfer features contained in X. We can clearly see that the biomarkers discovered across all four time-points are all longitudinally consistent. Visually, the brain heat-map images from Baseline to Month 24 look almost identical; this illustrates the power of the *l*_2,1_-norm regularization that provides our algorithm with the ability to identify longitudinally consistent biomarkers. This consistency is especially important from the clinical perspective. We find that the biomarkers identified by our method are strongly supported by previous research. For example, Mu *et al*.^19^ provide a review documenting how the hippocampus is affected by the early stages of AD; this part of the brain is one of the top-5 regions discovered by our model in Fig. 3. Van Hoesen *et al*.^30^ provide strong evidence that a severely damaged entorhinal cortex (Broadmann’s area 28) is observed in patients suffering from AD; the thickness of the entorhinal cortex is also identified by our method. Furthermore, Poulin *et al*.^23^ analyzed the impact of amygdala atrophy and determined that it was highly predictive of AD severity during the early clinical stages of AD; this finding is also supported by the FreeSurfer brain regions identified by our model.

#### SNP.

In Table 3 we rank the top-30 SNPs discovered by our algorithm. As we expect, the highest impact SNP discovered by our algorithm is rs429358; this SNP, known frequently as the APOE-*ε*4 allele, has been found^12^ to be highly predictive of early-onset AD. The authors’ note that approximately one third of the SNPs identified by our new method have previously been linked to AD; this further validates the utility of our approach in discovering well-known, as well as possibly-novel, AD biomarkers.

## 4. Conclusion

In this work we present a multi-block alternating direction method of multipliers approach to optimize the proposed new model that incorporates the *l*_2,1_ -norm, group *l*_1_-norm and trace-norm regularizations to discover important features contained in the ADNI dataset. This work illustrates a principled approach to combine multi-modal data using clinical time series data. The presented optimization algorithm is able to identify clinically relevant biomarkers and shows state-of-the-art predictive performance when jointly predicting the cognitive scores and diagnoses of ADNI participants.

## Figures and Tables

**Table 1.** Root mean-squared error values and standard deviations between the true and predicted RAVLT scores for the proposed method compared against an array of widely used machine learning algorithms. RAVLT scores vary between 0 and 74.

Model	RAVLTTOT	RAVLT30	RAVLT30-RECOG

*Linear*	4.19e11±7.20e11	1.06e12±1.34e12	8.85e11±1.06e12
*Ridge*	18.9±0.888	20.5±1.17	**19.6±0.872**
*Lasso*	19.4±0.913	21.1±1.29	20.0±0.957
*MLP*	19.2±0.961	20.7±1.25	19.8±1.05
*Our method*	**12.7±1.05**	**19.7±1.30**	19.8±0.928


**Table 2.** Multi-class *F*_*1*_ scores and their standard deviations, of the iterated five-fold cross validation experiments, for predicting the cognitive status of ADNI participants averaged over each time-point.

Model	*F*_1_(AD)	*F*_1_ (MCI)	*F*_1_ (HC)	*F*_1_ (All)

*Logistic*	0.265±0.0276	**0.500±0.0353**	0.313±0.0562	0.396±0.0299
*RandomForest*	0.325±0.0201	0.415±0.0466	0.401±0.0308	0.386±0.0325
*SVM*	0.289±0.0341	0.474±0.0450	0.363±0.0254	0.396±0.0286
*KNN*	0.330±0.0415	0.472±0.0524	0.410±0.0388	0.420±0.0332
*MLP*	0.312±0.0588	0.475±0.0523	0.341±0.0737	0.400±0.0366
*ElasticNet*^4^	0.255±0.070	0.447±0.0485	0.405±0.0655	0.390±0.0284
*LinearSVM*^13^	0.308±0.038	0.448±0.0381	0.332±0.0364	0.378±0.0311
*Our method*	**0.496±0.0419**	0.415±0.0222	**0.477±0.0308**	**0.459±0.0125**


**Table 3.** The top-30 SNPs identified by our algorithm.

1. rs429358^11,12^	7. rs17477673	13. rs7894245	19. rs2994978	25. rs212525^6^
2. rs7870463	8. rs11218301	14. rs4310446^10^	20. rs6746923	26. rs17477827
3. rs9461735	9. rs11687624	15. rs439401^11^	21. rs1801133^9^	27. rs2177828
4. rs6139494	10. rs405509^11,16^	16. rs1556758	22. rs7945931^17^	28. rs7036781^14^
5. rs17561	11. rs17123514	17. rs2248478	23. rs4631890	29. rs2627641
6. rs749008^28^	12. rs10512186	18. rs6037894	24. rs4713432^14^	30. rs17209374
